# Poly I:C-priming of adipose-derived mesenchymal stromal cells promotes a pro-tumorigenic phenotype in an immunocompetent mouse model of prostate cancer

**DOI:** 10.3389/fcell.2023.1145421

**Published:** 2023-11-22

**Authors:** Cosette M. Rivera-Cruz, Shreya Kumar, Marxa L. Figueiredo

**Affiliations:** Department of Basic Medical Sciences, Purdue University, West Lafayette, IN, United States

**Keywords:** mesenchymal stromal cells, adipose-derived mesenchymal stromal cells, priming, polarization, toll-like receptors, prostate cancer

## Abstract

**Introduction:** Mesenchymal stromal cells (MSC) are envisioned as a potential cellular vehicle for targeted cancer therapies due to their tumor tropism and immune permissiveness. An obstacle in their use is the duality in their interactions within tumors, rendering them pro-tumorigenic or anti-tumorigenic, in a context dependent manner. MSC preconditioning, or priming, has been proposed as a strategy for directing the effector properties of MSC at tumor sites.

**Methods:** We primed human MSC derived from adipose tissues (ASC), a clinically advantageous MSC source, utilizing toll-like receptor agonists. Subsequently, we explored the consequences in tumor progression and transcriptome upon the interaction of tumor cells with primed or unprimed ASC in an *in vivo* model of prostate cancer, the second most common cancer and second leading cause of cancer related death in men in the USA.

**Results and discussion:** In the studied model, poly I:C-primed ASC were found to significantly accelerate tumor growth progression. And while unprimed and LPS-primed ASC did not exert a significant effect on tumor growth at the macroscopic level, gene expression analyses suggested that all treatments promoted distinct modulatory effects in the tumor microenvironment, including altered modulation of angiogenesis, and immune response processes. However, the effects resulting from the collective interaction across these processes must be sufficiently skewed in a pro-tumorigenic or anti-tumorigenic direction for evidence of tumor progression modulation to be detectable at the macroscopic level. Our study highlights potential MSC-tumor microenvironment interactions that may be leveraged and should be considered in the development of cancer therapeutics utilizing MSC.

## 1 Introduction

Prostate cancer (PrCa) is the most common cancer and second leading cause of cancer related death in men in the United States of America (USA), expected to have accounted for 29% of new cancer cases and 11% of cancer deaths in men in the USA in 2023 (Siegel et al., 2023). Similarly to other cancers, despite many advances in treatments, a significant challenge to efficiently treat this disease is encountered once PrCa presents at an advanced stage, i.e., invasive adenocarcinoma, where the disease has started to spread outside of the prostate. For these cases, the traditional treatment course initially includes androgen deprivation therapy (ADT). Unfortunately, ADT can result in the selection of tumor cell clones that are no longer dependent on normal levels of androgens and can continue growing despite ADT. This transition is estimated to occur in 10%–20% of patients within approximately 5 years of follow-up (Kirby et al., 2011). PrCa that has progressed to this stage is termed castration resistant prostate cancer (CRPC), and the mean survival for patients with this stage of disease is 14 months. Challenges to therapeutic modalities outside of ADT often involve targeting inefficiency and limited dose tolerance due to toxicity, thus further exploration and development of therapeutics is needed, with an interest in the field on multimodal therapeutics and development of new targeting strategies for more efficient and less toxic therapeutic delivery (Chang et al., 2014; Terlizzi and Bossi, 2022).

Mesenchymal stromal cells (MSC) have been proposed as a potential cellular vehicles for targeted cancer therapies (Nowakowski et al., 2016). This is owing to an inherent tropism for the cells towards tumors and for their immune permissive phenotype, characteristics that are considered promising for enhancing therapeutic delivery while avoiding potential side effects. Also, rapid clearance can be avoided relative to other therapeutics such as triggered by chemotherapies and oncolytic viruses (e.g. an immune response) when administered systemically in the absence of a carrier. Further, MSC can interact with the tumor microenvironment (TME) by means of immunomodulation, modulation of metabolic processes, angiogenesis and extracellular matrix (ECM) deposition, thus having the potential of acting as effector cells beyond their roles as treatment delivery platforms. However, a duality in the interaction of MSC with tumors might render them pro-tumorigenic or anti-tumorigenic, in a context dependent manner (Norozi et al., 2016). These observations thus have raised interest in the development of strategies to enhance and/or consistently ‘guide’ MSC towards an anti-tumorigenic potential (Vicinanza et al., 2022). In 2010, a paradigm to MSC activation was proposed by which priming of BM-MSC with ligands of TLR4 or TLR3 yielded opposite cellular functional phenotypes (Waterman et al., 2010). More specifically, TLR4 stimulation by lipopolyssacharide (LPS) yielded a pro-inflammatory MSC phenotype, whereas TLR3 priming with polyinosinic:polycytidylic acid (polyI:C) rendered these cells immunosuppressive. These distinct phenotypes were evaluated in the context of an ovarian cancer *in vivo* model and the different TLR agonists were reported to exert anti-tumorigenic (LPS-primed) or pro-tumorigenic (poly I:C-primed) roles, respectively (Waterman et al., 2012).

While BM-MSC remain the most commonly studied MSC subtype, and were the focus of the MSC polarization paradigm reports, MSC derived from other tissues may provide advantages for clinical use such as higher relative abundance and differential functional potential (Strioga et al., 2012). In our previous work (Rivera-Cruz and Figueiredo, 2023), we had identified phenotypical and functional consequences of TLR priming on ASC that partially resembled those previously reported for BM-MSC (Waterman et al., 2010). In our assessment, LPS-priming of ASC rendered them immunostimulatory in immune killing assay coculture assays in the presence of human prostate adenocarcinoma cells and stimulated peripheral blood mononuclear cells. Understanding that tumorigenesis is a complex process involving many other TME components and other supportive tissues, we aimed to evaluate the consequences of TLR-priming in an immunocompetent model of androgen independent prostate cancer by assessing changes in tumor progression, gene expression and immune composition of tumors treated with TLR-primed ASC.

## 2 Materials and methods

### 2.1 Cell culture

Human ASC from a male Caucasian donor were obtained from Obatala. Cells were obtained from the vendor after characterization (e.g., surface marker expression profile, differentiation potential) and de-identification (Supplementary File S1). ASC were cultured on fibronectin-coated plasticware in modified media (ASC media) (Shearer et al., 2016). At passage 4, cells were transduced with a lentivirus to express renilla luciferase (LV-Rluc-Puro; G&P Bioscience, Santa Clara, CA) at MOI 2, using a ViraDuctin Lentivirus Transduction Kit (Cell BioLabs, San Diego, CA). Selection for transduced cells was performed for 2 weeks by supplementing growth media with 1 μg/mL of puromycin (Sigma-Aldrich). Mouse TRAMP-C2 (TC2) cells are advanced prostate adenocarcinoma cells (ATCC, CRL-2731), derived from a primary tumor in the prostate of Probasin-SV40-T antigen (TRAMP) adult C57/BL6 male mouse (Foster et al., 1997). Our group has further transduced TC2 with a lentivirus to express activated H-ras G12V and mouse androgen receptor (TC2R) (Zolochevska et al., 2013), observing significantly more rapid growth of these cells relative to the parental TC2 (Umbaugh et al., 2017). The TC2R cells serve as a model of aggressive PrCa to be examined in C57/BL6 immunocompetent mice. These cells were maintained in DMEM-F12 supplemented with 10% FBS and 1% Antibiotic-Antimycotic (Anti-Anti, Gibco).

### 2.2 ASC TLR priming

ASC were thawed and expanded as described above. After expansion, cells were seeded in 6-well tissue culture plates (not coated with fibronectin) at a density of 3 x 10^5^ cells/well and allowed to attach overnight in ASC media. ASC were primed *ex vivo* for 1 h in assay media containing DMEM-F12 (Corning) supplemented with 16.5% FBS (ATCC), 1% Anti-Anti. Unprimed MSC, incubated in base assay media, were used as control. TLR agonists were added to the base assay media as follows: Poly I:C 1 μg/mL (1:1 mix of high molecular weight (HMW) and low molecular weight (LMW) poly I:C, Invivogen, San Diego, CA), or LPS 10 ng/mL (Millipore-Sigma, Urbana, IL) (Waterman et al., 2010). After the completion of the hour, cell cultures were washed twice with 1x Dulbecco’s Phosphate Buffered Saline (Gibco), lifted by trypsinization and comixed with PrCa cells as described below for administration into the mice.

### 2.3 *In vivo* assessment of tumor progression

Animal care and procedures were performed in accordance with the Purdue Animal Care and Use Committee Institutional Review Board and the Laboratory Animal Program (LAP). All mice utilized in these experiments were housed in the Purdue LAP Facilities, where overall changes in mice health and behavior were monitored daily by facility technicians and veterinary staff. Tumors were induced in 10-week-old C57BL/6J male mice (Envigo) by subcutaneous injection of 1.25 x 10^5^ TC2R cells +1.25 x 10^3^ TLR-primed (or unprimed) ASC in sterile saline into their flanks (dorsolateral) while under isoflurane anesthesia. Mice injected only with TC2R cells were utilized as controls. Mice were randomly assigned to groups (n = 11; between two independent experiments), with at least one representative of each group per cage. The tumor growth was monitored over time utilizing vernier caliper measurements, in a blinded fashion. Tumor volume was calculated utilizing the following equation:

$$Tumor\;volume\left({mm}^{3}\right)=\frac{\pi }{6}\times (\text{larger diameter}\left(\text{mm}\right))\times {\left(smaller\;diameter\left(mm\right)\right)}^{2}$$

The experimental endpoint (19–21 days) was selected based on when at least one mouse reached the maximum allowable tumor burden in the study (tumor length of 20 mm).

### 2.4 Tissue collection

Mice were humanely euthanized by CO_2_ in several cohorts (through day 21) when the largest tumors within the experiment reached 20 mm in its their largest diameter. Tumor segments were collected in 10% buffered formalin and RNAlater (ThermoFisher, Mt Prospect, IL) and stored according to manufacturer’s protocols for later evaluation.

### 2.5 RNA isolation

Total RNA was isolated from timepoint-matched (day 20) tumors (n = 3 per treatment group) that had been preserved in 1 mL RNAlater, following manufacturer’s protocols, then RNAlater removed and dried tissues kept at −80°C. Briefly, tumors were immersed in 600 µL of RLT buffer +10 µL of β-Mercaptoethanol, and homogenized with a PRO200 homogenizer (MidSci, ValleyPark, MO, United States) in three brief pulses of 10–15 s each at a mid-power. Lysates were then processed using a Qiagen RNAeasy kit (Qiagen).

### 2.6 RNA-seq analysis

Sample processing for RNA sequencing was performed by LC Sciences (Houston, TX, United States), with analysis support. Briefly, a poly (A) RNA sequencing library was prepared following Illumina’s TruSeq-stranded-mRNA protocol. RNA integrity was checked with Agilent Technologies 2100 Bioanalyzer. Poly (A) tail-containing mRNAs were purified using oligo- (dT) magnetic beads with two rounds of purification. After purification, poly (A) RNA was fragmented using divalent cation buffer in elevated temperature. Quality control analysis and quantification of the sequencing library were performed using Agilent Technologies 2100 Bioanalyzer High Sensitivity DNA Chip. Paired-ended sequencing was performed on Illumina’s NovaSeq 6000 sequencing system. For transcripts assembly, cutadapt (Martin, 2011) and perl scripts in house were used to remove the reads that contained adaptor contamination, low quality bases and undetermined bases. Sequence quality was verified using FastQC (http://www.bioinformatics.babraham.ac.uk/projects/fastqc/), and HISAT2 (Kim et al., 2015) was used to map reads to the *Mus musculus* genome (ftp://ftp.ensembl.org/pub/release-101/fasta/mus_musculus/dna/). Reads were assembled using StringTie (Pertea et al., 2015), then transcriptomes were merged to reconstruct a comprehensive transcriptome using perl scripts and gffcompare. StringTie and ballgown (http://www.bioconductor.org/packages/release/bioc/html/ballgown.html) were used to estimate transcript expression levels. StringTie and ballgown (http://www.bioconductor.org/packages/release/bioc/html/ballgown.html) were used to estimate levels of expression of all transcripts. StringTie (Pertea et al., 2015) was used to perform expression level for mRNAs by calculating FPKM. Pearson correlation analysis was utilized to determine outliers within treatment groups, with R < 0.9 considered an outlier. mRNAs differential expression analysis was performed by R package DESeq2 (Love et al., 2014) between two different groups (and by R package edgeR (Robinson et al., 2010) between two samples). The mRNAs with the parameter of *p*-value below 0.05 and absolute fold change ≥1.5 were considered differentially expressed mRNAs. Metascape (Zhou et al., 2019) the Express Analysis was utilized to identify by ontology assessment potential cellular processes and pathways associated with the differential gene expression observations. Metascape identifies all statistically enriched terms and calculates accumulative hypergeometric *p*-values and enrichment factors used for filtering. The remaining significant terms are hierarchically clustered into a tree based on Kappa-statistical similarities among their gene memberships and a 0.3 kappa score is applied as the threshold to cast the tree into term clusters.

### 2.7 Immune cell profiling analysis using RNA-seq data

For a general analysis of the immune landscape, we utilized ImmuCellAI (Immune cell abundance identifier) (Miao et al., 2021) and TIMER2.0 (Li et al., 2020) to obtain estimated immune cell profiles, as well as broad microenvironment, immune, stromal, or infiltration scores. ImmuCell AI estimates the abundance of several immune cell (sub) types utilizing gene expression data and based on reference expression profiles and marker gene sets curated from pure immune cell types, using a ssGSEA enrichment score for the expression deviation profile for each cell type. TIMER2.0 uses the immunedeconv package (Sturm et al., 2019) to estimate immune cell abundance using six algorithms described in Li et al. (2020), also taking tissue-type specific information into account (PRAD, prostate adenocarcinoma, for example) in order to improve the estimation accuracy. In combination, these tools provide a detailed immune cell profiling (>100 (sub) cell types), utilizing preprocessing and visualization of all the estimations together from recently characterized algorithms (including EPIC, quanTIseq and TIMER) in the comprehensive platform (Li et al., 2020).

### 2.8 IPA analysis

Ingenuity Pathways Analysis (IPA) (Qiagen) Core Analysis was utilized to investigate mechanistic networks, upstream regulators, and functions of differentially expressed genes identified by RNA-seq analysis for each treatment group comparison to the untreated (No ASC) control group. The IPA analyses performed included i) Graphical Summary, and ii) Mechanistic Networks. The Graphical Summary integrates significant upstream regulators, diseases, functions, and pathways while reducing redundancy. It includes the most significant Canonical Pathways by *p*-value, regardless of their z-score strength. Upstream regulators include genes, mRNAs, and proteins, with consideration of the differential gene expression magnitude. This summary is based on precomputed relationships between molecules, functions, diseases, and pathways obtained through a machine learning algorithm. The networks are created using a heuristic graph algorithm and content-based machine learning (Krämer et al., 2022). The Mechanistic Networks analysis connects upstream regulators that can work together to elicit the gene expression changes observed in a dataset, with cutoff *p* < 0.05 and activation z-score > 2.0 or < -2.0.

### 2.9 Statistical analysis

Statistical analysis of RNA-seq data was performed *via* R package DESeq2. For other analyses, GraphPad Prism version 9.3.0 for Windows (GraphPad Software, San Diego, California United States, www.graphpad.com) was used to calculate statistically significant differences among groups, using multiple *t*-test or ANOVA with *p*-value <0.05 considered significant. Statistical test or package used for each dataset is described in the corresponding figure legends.

## 3 Results

### 3.1 Poly I:C-primed, but not unprimed nor LPS-primed ASC promote prostate cancer tumor progression

**FIGURE 1:**
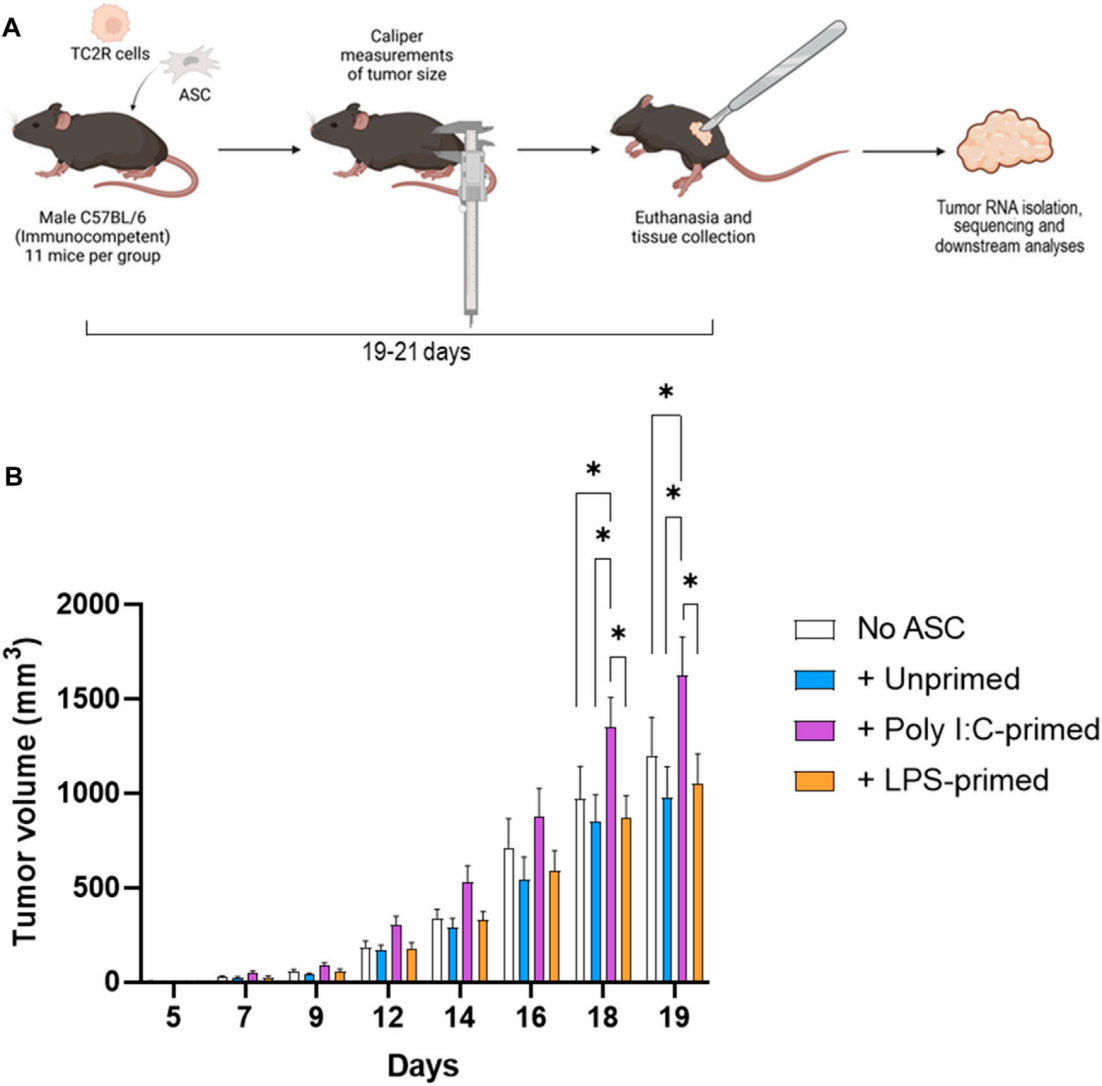
Tumor induction and monitoring. **(A)**. Diagram depicting the experimental workflow. Prostate cancer tumors (TC2R) were induced *via* co-injection of TLR-primed ASC and tumor progression was followed *via* caliper measurements over time. At day 19–21, mice were euthanized, and tumors were excised and processed for RNA sequencing as described in the methods section. **(B)**. Tumor volume time course. Data is presented as mean (n = 11, from two independent experiments) ± SEM. Groups included: No ASC (white bars), +Unprimed ASC (blue bars), +PolyI:C-primed ASC (purple bars), and +LPS-primed ASC (orange bars). Analysis was performed by a two-way ANOVA between treatment groups, and a *p*-value of <0.05 was considered significant (*).

To assess the effects of TLR-priming on ASC-mediated modulation of tumor progression, we evaluated the effect of the presence of these ASC in the prostate tumor growth rates in a murine model. For this purpose, syngeneic TC2R tumors were induced in immunocompetent mice, accompanied by a dose of either *ex vivo* TLR-primed ASC, unprimed ASC, or no ASC. Tumor sizes were monitored *via* caliper measurements for ∼3 weeks (Figure 1, Supplementary File S2). For these experiments, mice injected with tumor cells only (no ASC) were selected as a control group. While no significant differences in tumor growth were observed in unprimed ASC and LPS-primed ASC compared to the untreated controls, a significant increase in tumor growth relative to all other experimental groups was observed in mice that received poly I:C-primed ASC (Figure 1). At day 19 after tumor induction, this represented a 35% increase in tumor size in the poly I:C-primed ASC-treated relative to the untreated control (no ASC) group. No sporadic mouse deaths occurred in the duration of the described experiments. At day 19, five mice met our end-point tumor burden criteria (Treatment groups: one No ASC, one + LPS-primed ASC, and three + Poly I:C-primed ASC). These mice were euthanized on day 19 and the remaining mice were euthanized between days 19–21, following euthanasia guidelines based on tumor burden. The tumor growth dynamics observed suggested that administering unprimed or LPS-primed cells at these low ratios (1% ASC relative to starting tumor cell count), although physiologically significant in the context of a prostate tumor (Brennen et al., 2013), were not sufficient to induce a significant pro-tumorigenic nor anti-tumorigenic response in the studied model. On the other hand, poly I:C-primed ASC acted in a pro-tumorigenic manner, significantly worsening disease progression.

### 3.2 Differential gene expression in the tumors

**FIGURE 2:**
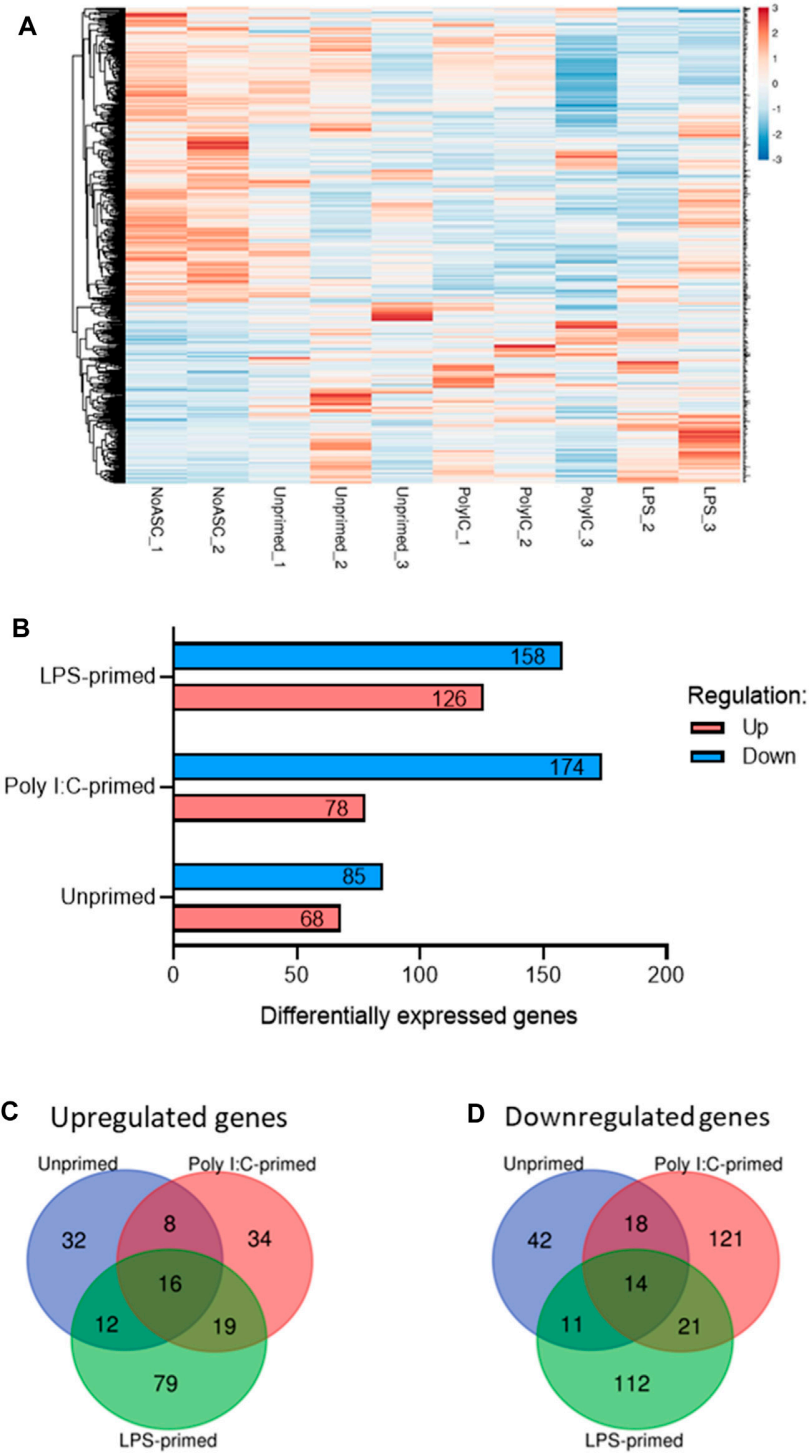
Summary of differentially expressed genes identified *via* RNA-seq of tumors. **(A)**. Heatmap of differentially expressed genes in one or more treatment group relative to No ASC controls generated *via* ClustVis (Metsalu and Vilo, 2015). Rows are centered and clustered using Euclidean distance and average linkage. Unit variance scaling was applied to rows from RPKM input values and values were plotted, with red representing higher relative expression and blue representing lower relative expression. **(B)**. Summary of up- (red) and downregulated (blue) genes for each treatment group relative to the untreated control tumors. Summary of overlap in genes upregulated **(C)** and downregulated **(D)** across treatment groups using Venn diagrams generated using the Van de Peer Lab Venn diagram tool (http://bioinformatics.psb.ugent.be/webtools/Venn/). Genes were considered differentially expressed if FC ≥ 1.5 or ≤ −1.5 and *p*-value <0.05 as determined by DESeq2 analysis (Love et al., 2014).

Having observed significant differences in tumor growth rates across treatment groups, we sought to investigate potential modes of action mediating these effects. For this purpose, RNA-seq analysis was performed in representative timepoint-matched tumor samples (n = 3) as described in section 3.2.6. RNA-seq analysis identified a total of 541 genes differentially expressed (Fold Change (FC) ≥ 1.5 or ≤ −1.5 and *p*-value <0.05) across one or more treatment groups relative to the No ASC control tumors (Figure 2). Changes in expression of 420 (77.6%) of these genes were found to be unique to a single treatment group, whereas 30 genes (5.5%) were found to be regulated in the same direction across all treatment groups relative to the No ASC controls (Figures 2C, D). No genes were regulated with opposite directionality across our treatment groups.

**FIGURE 3:**
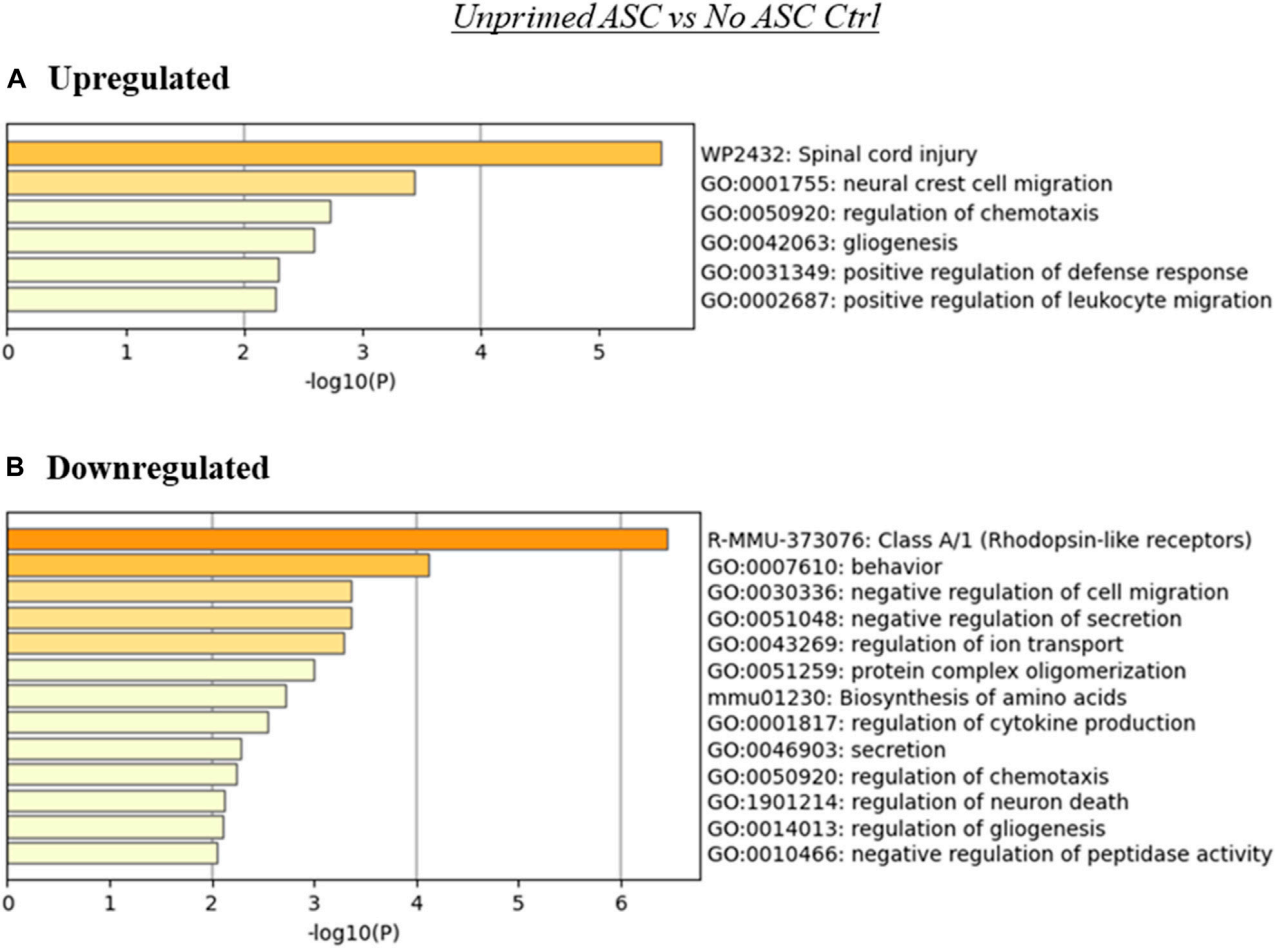
Summary of enriched terms identified in unprimed ASC treated tumors relative to untreated controls. Genes differentially expressed in unprimed ASC treated samples relative to untreated tumor controls were evaluated *via* Metascape enrichment analysis. Separate analyses were conducted for genes based on their regulation [**(A)** upregulated, **(B)** downregulated)]. Figures depict statistically significant enriched terms (-log10(p)) in a bar plot format, obtained from hierarchical clustering of processes based on Kappa-statistical similarities among their gene memberships.

**FIGURE 4:**
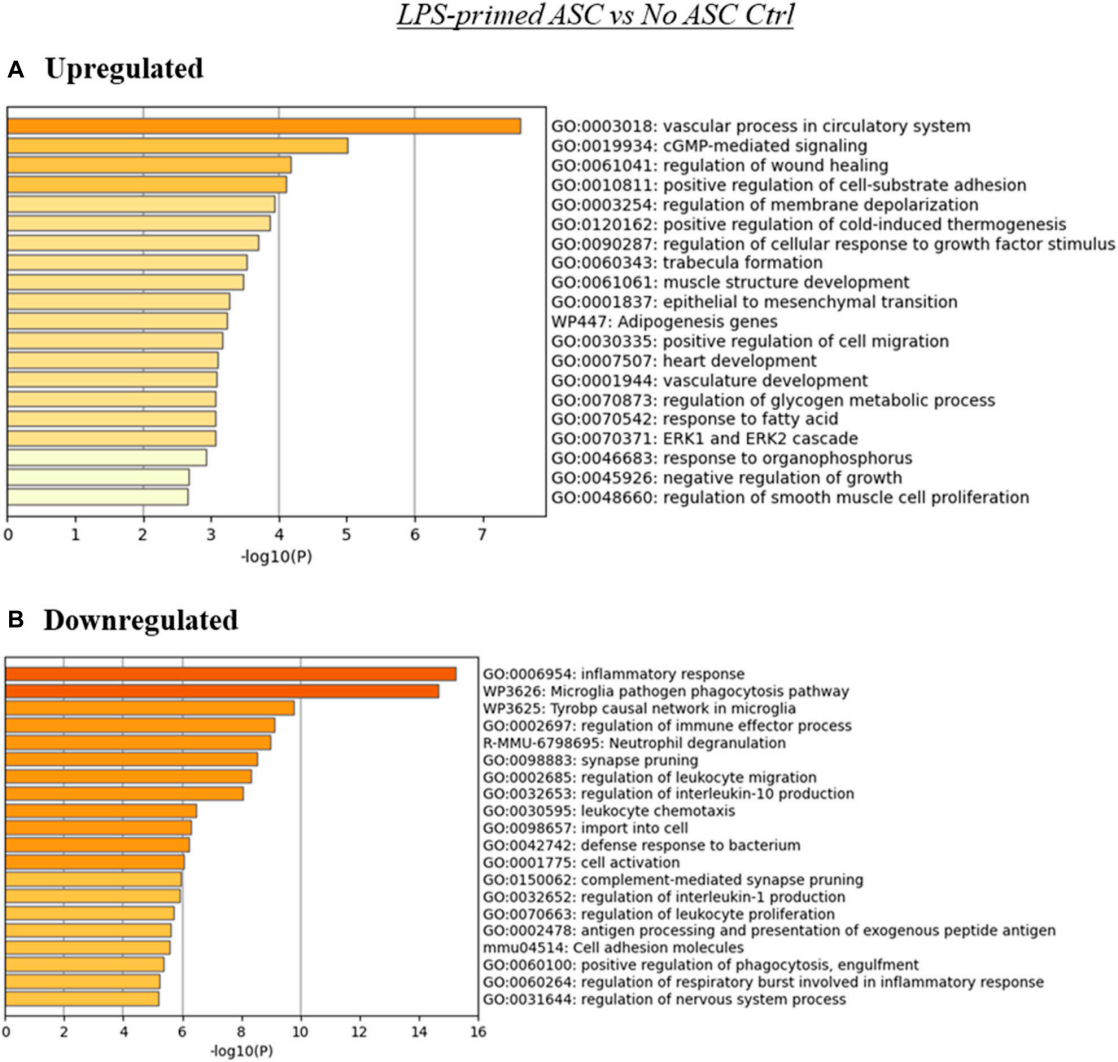
Summary of enriched terms identified in LPS-primed ASC treated tumors relative to untreated controls. Genes differentially expressed in LPS-primed ASC treated samples relative to untreated tumor controls were evaluated *via* Metascape enrichment analysis. Separate analyses were conducted for genes based on their regulation [**(A)** upregulated, **(B)** downregulated)]. Figures depict statistically significant enriched terms (-log10(p)) in a bar plot format, obtained from hierarchical clustering of processes based on Kappa-statistical similarities among their gene memberships.

MSC are capable of interacting with tumor cells and components of the TME through various mechanisms, including immunomodulation *via* cytokine secretion and cell-to-cell contact (Rivera-Cruz et al., 2017), ECM deposition (Hass, 2020), and metabolic reprogramming (Pers et al., 2021). To gain some understanding of the consequences of the differential expression of genes observed across our samples in the context of cellular processes and pathways, we performed ontology enrichment analysis *via* Metascape for genes differentially expressed relative to the untreated tumor controls, within each treatment group (Figures 3, 4, Supplementary File S3). As expected, based on the overlap in the differentially expressed genes observed across samples (Figures 2B, C), regulation of some processes was shared across two or more treatment groups. Among these were processes associated with cell migration and chemotaxis, and immunity.

While no significant differences in tumor progression rate were noted in tumors treated with unprimed or LPS-primed ASC relative to the untreated controls (Figure 1), our RNA-seq data suggests that there were several significantly altered processes in these tumors (Figures 3, 4). The presence of unprimed ASC in TC2R tumors was found to upregulate genes associated with enrichment in nervous system processes such as migration and gliogenesis, and immune related processes such as the positive regulation of leukocyte migration (Figure 3A). On the other hand, terms enriched within downregulated genes included Class A/1 (Rhodopsin-like receptors) and processes related to cell secretion, among other neural and immunity related processes (Figure 3B).

The analysis using the genes upregulated in the LPS-primed ASC treated tumors relative to control (no ASC) TC2R tumors produced several enriched terms, with the most significant hits aligning with processes associated with vasculature and cGMP-mediated signaling (Figure 4A). Downregulated genes for this treatment group were primarily enriched in terms related to inflammatory and immune processes (Figure 4B).

**FIGURE 5:**
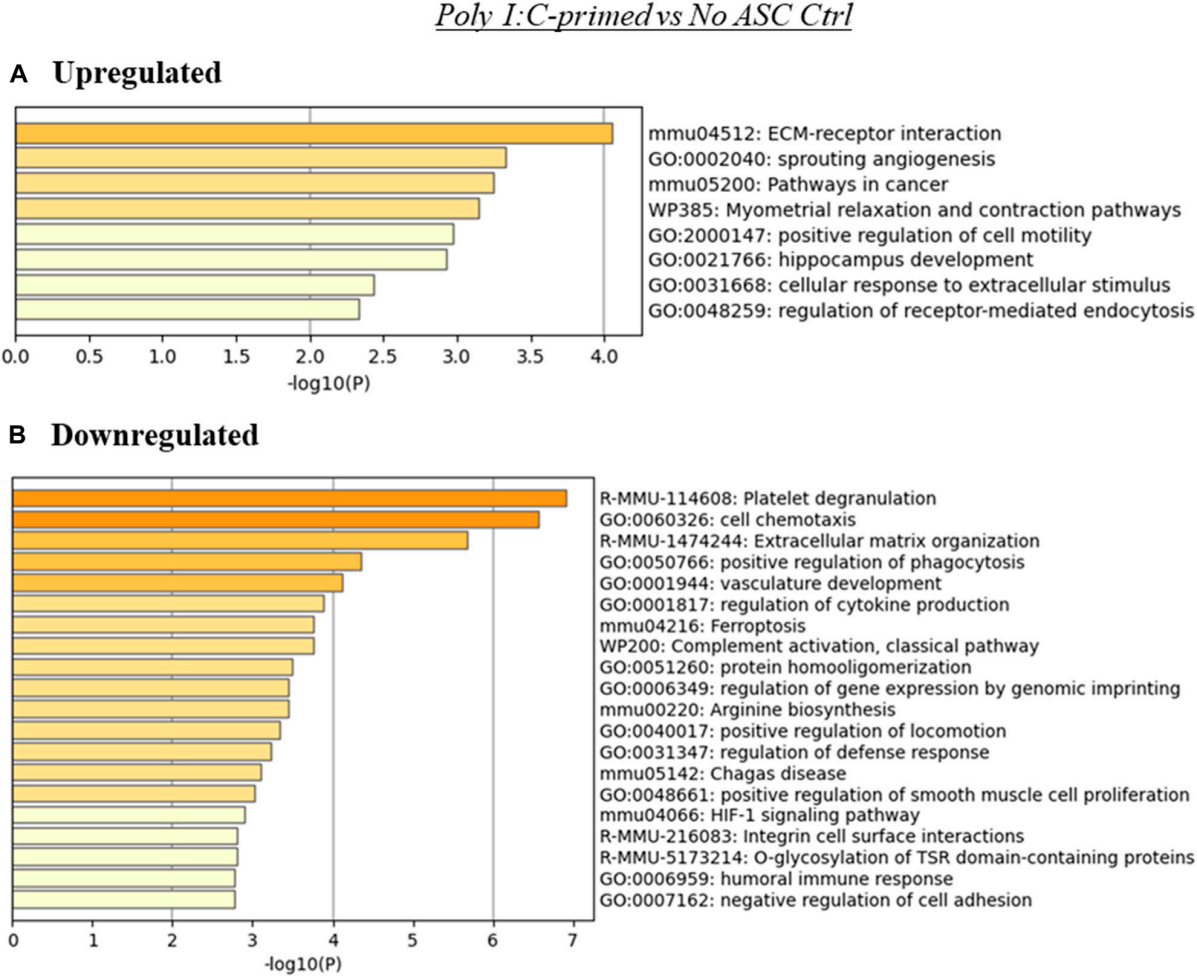
Summary of enriched terms identified in poly I:C-primed ASC treated tumors relative to untreated controls. Genes differentially expressed in poly I:C-primed ASC treated samples relative to untreated tumor controls were evaluated *via* Metascape enrichment analysis. Separate analyses were conducted for genes based on their regulation [**(A)** upregulated, **(B)** downregulated). Figures depict statistically significant enriched terms (-log10(p)) in a bar plot format, obtained from hierarchical clustering of processes based on Kappa-statistical similarities among their gene memberships.

Since poly I:C ASC presence in tumors promoted their growth rate, this may suggest that alterations to the TME effected by the administered primed ASC collaborate with the already tumor permissive environment that develops at these tumor sites. The analysis using the genes upregulated in the Poly I:C-primed ASC group relative to control (no ASC) tumors indicated, for the upregulated genes, an enrichment of terms related to ECM-receptor interactions, sprouting angiogenesis and pathways in cancer, among others (Figure 5A). On the other hand, the genes downregulated in this treatment group relative to control were enriched in several immune-related and cell adhesion or ECM-related processes, among others (Figure 5B).

When evaluating ontologies relevant to differential gene expression between TLR-primed and unprimed treated samples (Supplementary File S4), processes involving regulation of cell migration, cytokine production and immunomodulation emerged as the most significantly enriched. Collectively, these findings suggested that administering ASC in either a primed or unprimed status can significantly modulate processes within tumors relating to the communication between tumor cells with the microenvironment, as well as with the immune system. A common target of this modulation are immune processes, although the nature of these changes may diverge across different groups depending on the ASC priming modality. These modulated processes likely act in conjunction, and the interplay among the many molecular processes influence the overall effects detected at the macroscopic level in altered tumor progression.

### 3.3 Estimation of altered immune composition in the tumors

Given that several immune processes were shown to be enriched within our treatment groups relative to the untreated tumor controls, and that some of these changes diverged across ASC treatment groups, we sought to evaluate potential differences in immune composition across these groups. For this purpose, we utilized two web-based tools with algorithms that permit the estimation of immune components within samples based on bulk RNA-seq analysis, ImmuCell AI and TIMER2.0.

**FIGURE 6:**
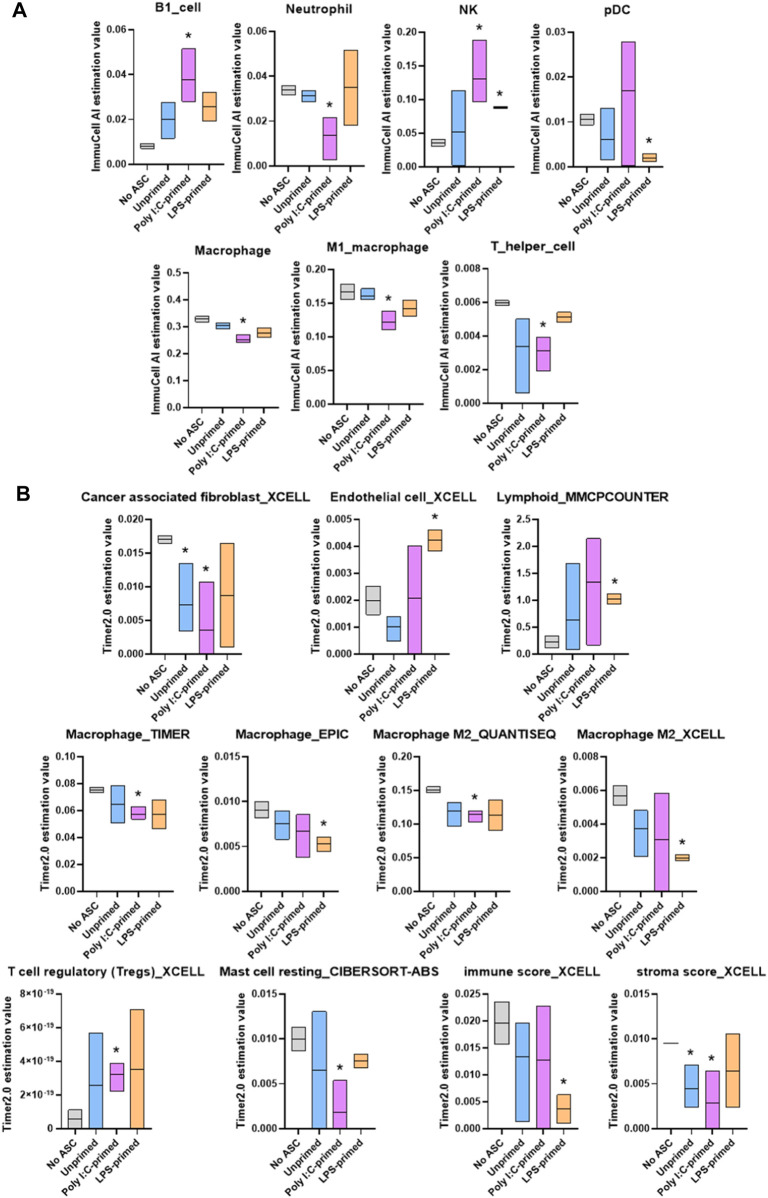
Summary of significant differences in immune cell abundance between treatment groups. Immune cell abundance in tumor samples were estimated from bulk RNA-seq data using ImmuCell AI **(A)** and TIMER 2.0 **(B)** algorithms. Cell populations with significant differences in abundance relative to untreated controls in at least one treatment group are included in the figure. Data presented as abundance estimation value in min-to-max box plots with line representing the mean. Analysis was performed by multiple t-tests, comparing each treatment group to the No ASC controls, *p*-value <0.05 was considered significant (*).

Through these analyses, we identified significant changes in several cell populations relevant to immune response in tumors (Figure 6). Namely, tumors treated with unprimed ASC showed significantly lower cancer associated fibroblasts and stromal scores relative to control (no ASC) tumors. Poly I:C-primed ASC treated tumors displayed a significant increase in B1 cells, NK cells and Tregs, whereas neutrophils, macrophages, T helper cells, cancer associated fibroblasts, resting mast cells, and stroma score values were decreased in these tumors relative to controls (no ASC). In the case of the LPS-primed ASC treated group, significantly higher abundance scores were obtained for NK, endothelial, and lymphoid cells, yet pDC, macrophage, and immune scores were lower relative to the control tumors (no ASC). Together this data suggested that administration of ASC into tumors even at low percentages (1% of initial tumor cell count) can significantly alter the tumor immune composition, regardless of their priming status. These immune changes by unprimed or LPS-primed ASC, however, were insufficient to tilt the TME to either a pro-tumorigenic or an anti-tumorigenic phenotype. In the case of poly I:C-primed ASC treated tumors, it is likely that the differences in tumor immune composition may have contributed to the increased tumor growth rate observed.

### 3.4 Regulatory networks by Ingenuity pathway analysis (IPA)

**FIGURE 7:**
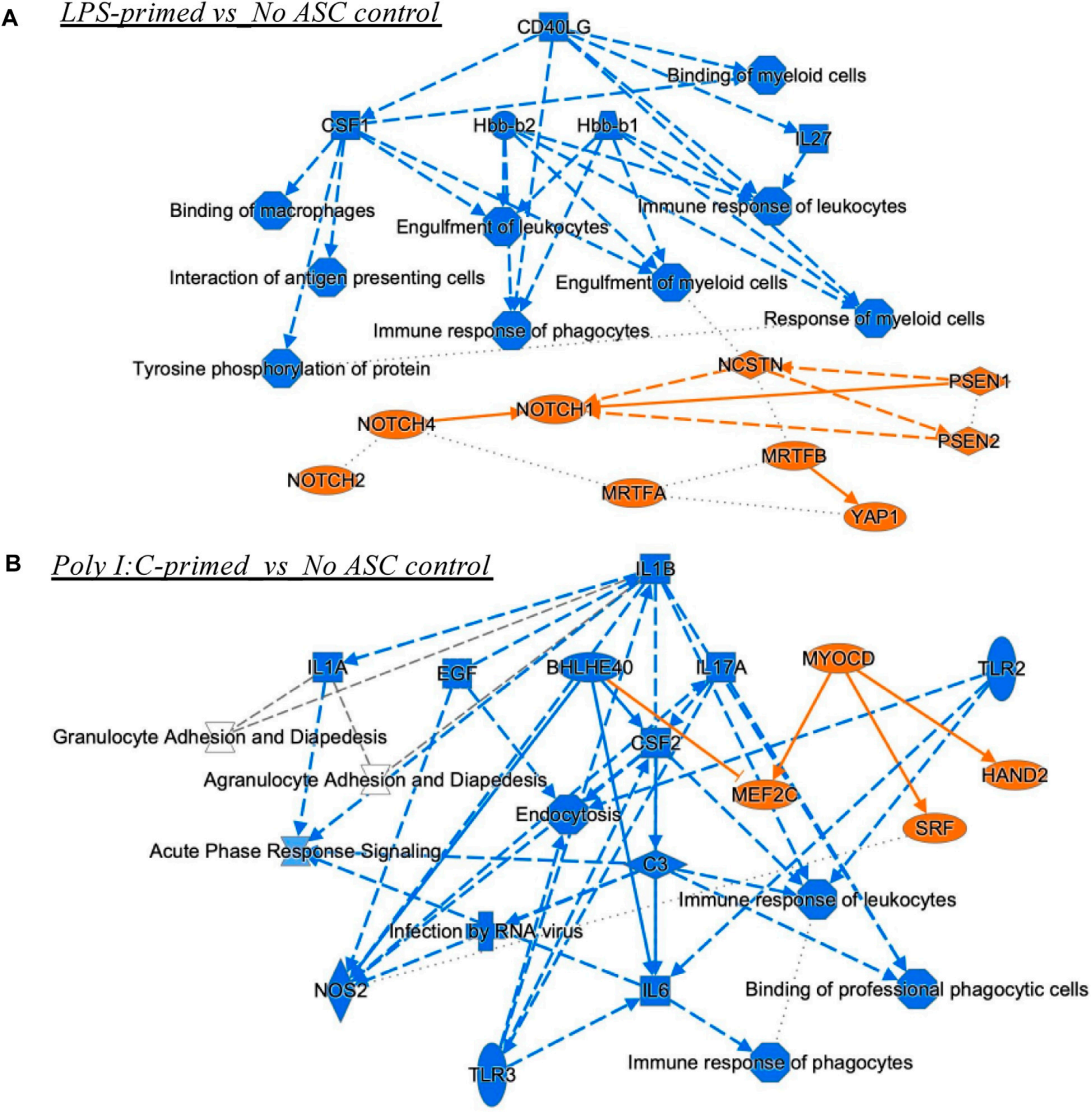
Graphical summary of IPA analyses. These include Upstream Regulators and Diseases and Functions analyses for comparisons LPS-primed versus No ASC control **(A)**, and PolyI:C-primed versus No ASC control **(B)** that had significant z-scores (lowest B-H corrected *p* values). The unprimed ASC group did not have enough upstream regulators or diseases and functions for connectivity in the graphical summary analysis. Orange, predicted activation, Blue, predicted inhibition. Solid lines, direct relationship or interaction; Dotted lines, inferred relationship, or indirect interaction.

A set of additional analyses was performed using IPA to obtain some insight into the potential mechanisms underlying the observed differences in tumor growth rate by the differently primed ASC populations relative to the no ASC control. The graphical summary analysis provided an overview of the major biological themes emerging from the most significant entities detected in the datasets to help illustrate how these themes connected to each other. For the LPS-primed group, several processes and molecules were predicted as inhibited, in particular, upstream regulators CD40LG (CD40 ligand), colony stimulating factor (CSF)1, Hbb (hemoglobin subunit beta)-b1/b2, and interleukin (IL)-27, with connecting effects of reduced response from phagocytes and other myeloid cells. Interestingly, NOTCH (notch receptor) related molecules were predicted as activated regulators, connecting at least to the engulfment of myeloid cells function (Figure 7A). The poly I:C-primed ASC group showed a strong influence of inhibition of IL-1B (and IL-1A), epidermal growth factor (EGF), IL-17A, CSF2, IL-6, and TLR3, connecting to predicted reductions in the immune response of phagocytes and leucocytes, and APC binding. The activated regulators included MEF2C (myocyte enhancer factor 2C), through BHLHE40 (basic helix-loop-helix family member E40) inhibition and from MYOCD (myocylin) activation, and also activation of SRF (serum response factor) and HAND2 (heart and neural crest derivatives expressed 2) (Figure 7B).

**FIGURE 8:**
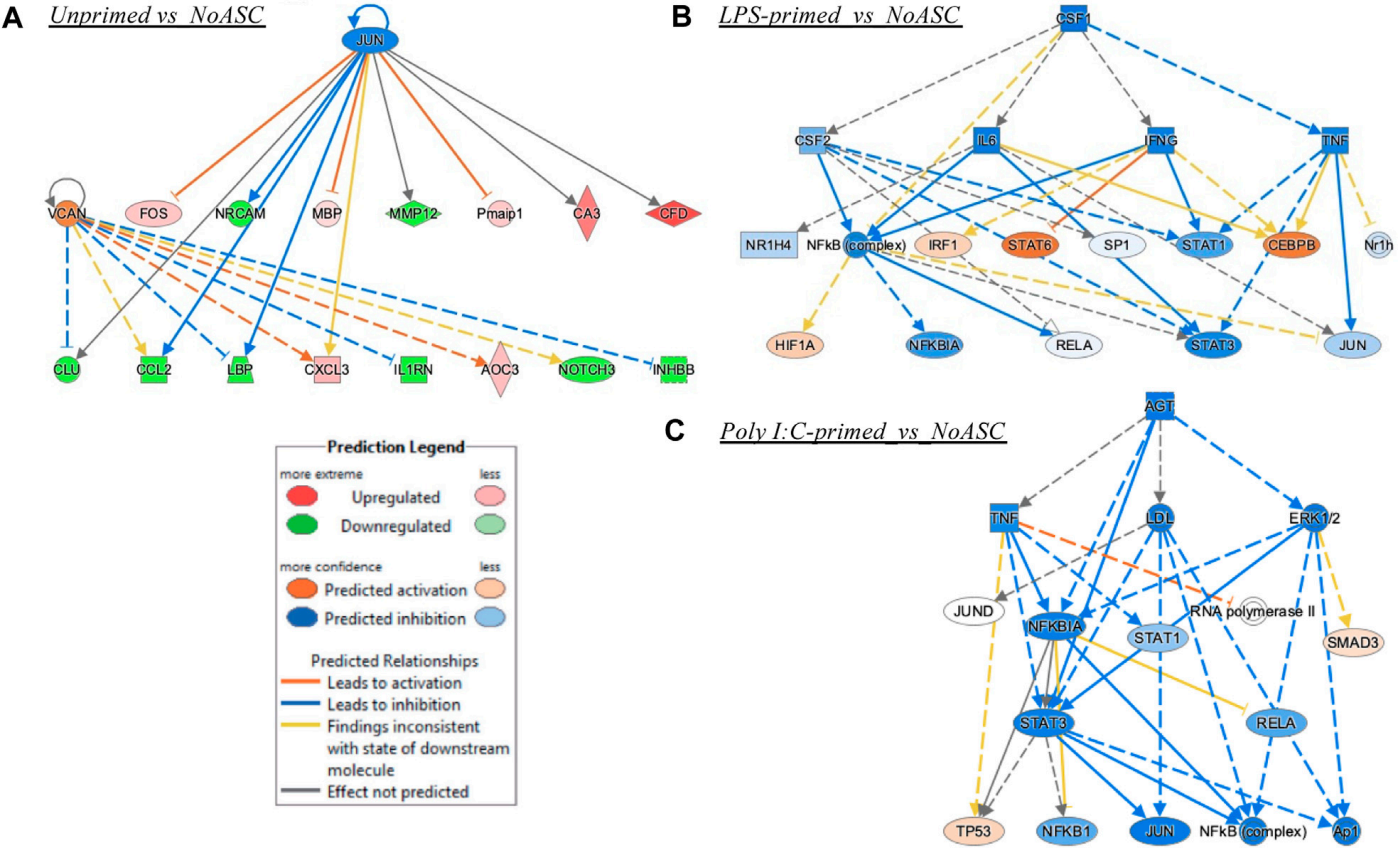
Mechanistic networks formed by each treatment group. **(A)** Unprimed ASC vs. No ASC control treatment of TC2R tumors, **(B)** LPS-primed ASC vs. No ASC control; **(C)** Poly I:C-primed ASC vs. No ASC control. Prediction Legend indicates the directionality of predicted activation of inhibition, whereas the relationships among the nodes are illustrated by a variety of color lines.

Finally, the unprimed ASC population did not have enough connectable entities with sufficiently high or low z-scores to meet the cutoffs. Yet, the unprimed ASC treated tumors had VCAN (Versican) as an activated upstream regulator with a z-score of 1.4 and an inhibited regulator JUN protooncogene with a z-score of −1.5, both with significant values for the multiple testing corrected *p*-value (B-H or Benjamin-Hochberg). For the unprimed ASC group, JUN inhibition along with VCAN activation connect to the potentially anti-tumorigenic mechanisms of reduced matrix metallopeptidase (MMP) 12 activation, and a decrease in C-C Motif Chemokine Ligand (CCL) 2, NOTCH3, and other nodes of interest that can be pursued in future studies (Figure 8A). For the LPS primed ASC group, Signal Transducer And Activator Of Transcription (STAT) 3 was ultimately inhibited, and this could represent a mechanism of anti-tumorigenesis (Figure 8B). For the poly I:C-primed ASC group, an interesting mechanistic network differed from the LPS group in a predicted inhibition of Mitogen-Activated Protein Kinase 3/1 (ERK1/2), and in a stronger predicted inhibition of JUN, along with a predicted activation of Tumor Protein 53 (TP53), a key tumor-suppressor gene (Figure 8C).

## 4 Discussion

With the emergence of an interest in the use of mesenchymal stromal cells for anti-cancer therapeutics, there is an increased need to better understand the mechanisms by which MSC may be regulated to exert anti-tumorigenic functions and prevent their pro-tumorigenic activities. Among these strategies, pre-conditioning by *ex vivo* stimulation with TLR ligands has been proposed to promote polarized pro- or anti-inflammatory MSC phenotypes with pro- or anti-tumorigenic properties, respectively. This phenomenon, however, has remained largely unexplored in MSC from alternative tissue sources beyond BM-MSC, for example, ASC, which may provide clinical advantages because of culture characteristics that allow a faster acquisition of clinically relevant doses in reduced time.

In our previous work (Rivera-Cruz and Figueiredo, 2023), we identified similarities to the polarization phenotype occurring in ASC in response to TLR priming. However, our work was limited in that, in the functional evaluation of TLR-primed ASC within this study, we studied interactions between primed ASC, immune cells, and cancer cells in isolation. Tumorigenesis and tumor immunity are also influenced by more complex processes, such as angiogenesis, interactions with the tumor stroma, among others. To further our understanding of the consequences of TLR-priming in modulating the effect of ASC administration in prostate cancer tumors, we evaluated tumor growth rates and mRNA signatures of tumors treated with ASC of different priming statuses and compared them to those of untreated tumors.

By monitoring tumor size over time in our model, we identified a significant increase in the growth rate of tumors treated with poly I:C-primed ASC relative to all other treatment groups. These observations were consistent across two independent experiments for which pooled results are summarized in Figure 1. No significant differences were observed relative to the untreated control group, in the unprimed ASC nor the LPS-primed ASC treatment groups. These findings are interesting in that they partially recapitulate the effects of MSC-tumor interactions observed in the initial polarization reports, in that poly I:C induced a pro-tumorigenic action by the BM-MSC treatments (Waterman et al., 2012). Yet, the potentially anti-tumorigenic effects of LPS-primed cells reported by this group and suggested in our ASC *in vitro* experiments (Rivera-Cruz and Figueiredo, 2023) were not evident in these *in vivo* experiments. There are many potential reasons for these observed differences. When compared to the reports by Waterman *et al.,* potential contributors to these differences may include a distinct MSC tissue source, differences in cell administration route and dosing, and ultimately distinctions in the tumor models assessed, including tumor location and tumor type. To better understand potential mechanisms mediating these observed differences, we performed RNA-seq analysis on representative tumor samples from each treatment group. Interestingly, while no significant differences had been observed at the macroscopic level in unprimed ASC nor LPS-primed ASC treated tumors (Figure 1), significant differences were observed between these groups and the untreated controls at the level of gene expression (Figure 2). While our heatmap displayed variability across samples within the same ASC-treated groups, indicating that a degree of heterogeneity may be maintained in the ASC population despite priming, many of the differentially expressed genes were uniquely identified in their respective treatment groups (Figures 2C, D). This suggests that the administration of these cells might induce treatment-specific differences in the tumors. However, these differences may not always be sufficient to potentiate altered tumor growth rates detectable at the macroscopic level.

For the unprimed and LPS-primed ASC treatment groups, among the enriched terms found by the Metascape ontology assessment based on differentially expressed genes were terms related to nervous system, cell migration, and immune system related processes, among others (Figures 3, 4). Intriguingly, enrichment of terms related to these categories was identified to some extent in both the upregulated and downregulated genes for these groups, suggesting that there is not a clear directionality of these changes. This, however, matches our observation in that no evident (nor significant) effects were observed in tumor development over time. These enriched categories are however interesting in that they encompass a variety of potential mechanisms for tumor progression modulation relevant to prostate cancer. For example, in the case of nervous system related processes, in prostate cancer and other cancer types, neurogenesis and axon genesis associated with tumors and subsequent nerve-cancer crosstalk is understood to drive carcinogenesis (Silverman et al., 2021). Further, although prostate adenocarcinomas are of epithelial origin, neuroendocrine differentiation can occur following treatment, and it is understood to contribute to mechanisms of ADT resistance and worsening prognosis in prostate cancer (Kaarijärvi et al., 2021). These neuroendocrine cells are typically independent of androgens and have been shown to support the growth of androgen-sensitive adenocarcinoma even under androgen ablation (Jin et al., 2004). Potential roles on the induction of neuroendocrine differentiation have been attributed to cancer associated fibroblasts (CAF), a cell population that is believed to be able to originate from MSC (Kato et al., 2019). In the case of processes associated with cell migration, these processes may affect recruitment of other cell populations, such as immune cells, to the TME. In conjunction with other immune system related processes, these changes may contribute to differences in immune infiltration and response to the tumors. MSC are known to exert functions in immune recruitment and immunomodulation *via* cell-cell contact and soluble factors, and the nature of these interactions may be pro- or anti-tumorigenic depending on the context (Rivera-Cruz et al., 2017).

In the poly I:C-primed ASC treatment group, similarly to the other ASC-treated groups, several overarching categories of processes were modulated, some of which were enriched in both the upregulated and downregulated gene lists (Figure 5). Some examples included ECM-receptor interactions, angiogenesis, and cell motility-related processes. Since we detected significantly different effects in the tumor growth rate for the poly I:C ASC-treated group, this may suggest that any specific TME-related processes were sufficient to induce a more pro-tumorigenic phenotype in this group. ECM-related processes could be implicated, as these were among the terms enriched in both upregulated and downregulated genes. Dysregulation of ECM-related proteins, such as integrins, is implicated in modulation of tumor progression by roles in cell proliferation, cell adhesion, migration and angiogenesis (Venning et al., 2015). Development of new vasculature, or angiogenesis, is commonly considered a hallmark of cancer progression as the new vasculature provides access to a higher blood supply, enabling tumor cell proliferation (Hanahan, 2022). While targeting angiogenesis is of interest in the field of cancer therapeutics, this is a difficult task as angiogenesis signaling is highly redundant (Melegh and Oltean, 2019). While MSC have been reported to be able to have anti-angiogenic roles (Pan et al., 2019), more often they have been reported to exert pro-angiogenic properties within tumors, for example, through the secretion of angiogenic factors (i.e., VEGF) (Beckermann et al., 2008).

A few functional processes were differentially enriched in poly I:C-treated samples, which may have “tilted the balance” towards the more rapid tumor growth detected in our *in vivo* experiments. Among these, several immune processes were enriched in the list of downregulated genes in this treatment group. Examples of these processes included phagocytosis and humoral response. Interestingly, when evaluating the relative abundance of immune cell populations in these tumors (Figure 6), the poly I:C-primed ASC treated samples were found to contain significantly reduced phagocytic populations such as neutrophils and macrophages. We did, however, also detect a significant increase in B1 cells, which was unexpected in the context of the suppression of humoral response. Further supporting a more tumorigenic phenotype following the poly I:C ASC treatment, we also observed an increase in the abundance of regulatory T cells. While the effect of decreases in resting mast cells, stromal scores and CAFs, and increases in NK cell abundance might potentially promote an opposite immunomodulatory effect, for the latter, this would depend on the activation state, which is not assessed in this estimation. Interestingly, in a study looking at immune infiltration in prostate tumors comparing 190 normal prostate tissues to 537 PrCa samples (Wu et al., 2020), NK cells were found more abundantly in tumor tissues, with the majority of these cells found at a resting stage. Further, this group also found that PrCa tumors have a lower abundance of resting mast cells compared to normal tissues. These observations are concordant with our observations in immune cell estimates in poly I:C for these cell subsets and these at least in part may be correlated with disease progression. However, observations in regard to macrophage and neutrophil infiltrates by Wu et al. (2020) were dissimilar to our observations of decreased amounts in these populations within our tumor samples relative to control (no ASC).

The reduced response from phagocytes and other myeloid cells in the LPS primed group detected by IPA was consistent with detection of lower macrophages in the immune profiling data. Interestingly, NOTCH activation is associated with the promotion of macrophage activation towards an anti-tumor (M1) phenotype (Wang et al., 2010). This further supports the notion of potentially counteracting mechanisms being at play nullifying each other and somewhat maintaining a balance where a distinct pro-tumorigenic/anti-tumorigenic role cannot be clearly observed. Future studies can explore dosage changes or alterations to the priming protocol to further potentiate the roles of NOTCH signaling and other anti-tumorigenic consequences of LPS-primed ASC on tumor growth. The poly I:C group, paradoxically, showed a reduction in many regulators that are pro-inflammatory, yet impacted myeloid cell populations, consistent with the immune cell profiling data of reduced M1 macrophage abundance in tumors. The M1 macrophage is tumoricidal and as such, the reduction in that population through several potential mechanisms (CSF2 reduction may be one (Sielska et al., 2020)) can impact tumorigenesis. Some of the regulators affected by this treatment however could be implicated in anti-tumorigenic activities (*i.e.,* IL-6 (Jiang et al., 2011)), yet modulation identified in other regulators are potentially implicated in prostate cancer tumorigenesis, such as BHLHE40 (Dmitriev et al., 2015), and SRF (Prencipe et al., 2018). This once again suggests that modulatory effects of these treatments result in a tug-of-war of pro- and anti-tumorigenic actions yet poly I:C-primed ASC treatments used in these experiments were capable of “tilting the balance” of these interactions to a more pro-tumorigenic environment as suggested by the effects observed on tumor progression *in vivo.*

In addition to the mechanisms described earlier, it remains possible that the pro-tumorigenic potential of poly I:C-primed ASC may also be mediated by an increased abundance or survival of ASC at the tumor site. Although we did not assess ASC survival/abundance in the studied tumors, previous data from our group and others suggest that ASC are non-tumorigenic (Zolochevska et al., 2012) and that TLR-primed MSC are capable of modulating tumor growth without significantly inducing MSC colony formation or tumor formation potential (Waterman et al., 2012). However, the question of whether TLR-priming affects ASC survival and tumor-forming potential *in vivo* remains unanswered and may be the focus of future investigations. Furthermore, additional work is needed to functionally validate the mechanisms proposed in this study and to explore how various preconditioning parameters, dosages, and routes of administration can affect the complex balance of MSC-tumor interactions (Otsu et al., 2009; Zheng et al., 2016). While our study provides a solid foundation for informing subsequent investigations of MSC-tumor interactions, it is important to note that, in this study, primed-ASC were co-administered with cancer cells. This co-administration may not closely resemble the clinical use of these cells. Therefore, we should in future studies explore other contexts that more closely resemble clinical settings, such as systemic administration to tumor-bearing mice.

## 5 Conclusion

Taken together our data suggest that TLR-priming of MSC may differentially affect the tumor progression modulation roles of ASC. Mediating these roles are likely processes involving angiogenesis, immunomodulation, and others such as nervous system-related processes and chemotaxis, with roles that may influence pro- or anti-tumorigenic actions. However, the effects resulting from the collective interaction across these processes must be sufficiently skewed in one of these directions for evidence of tumor progression modulation to be detectable at the macroscopic level. An example was the observation made from the analyses of the tumors treated with poly I:C-primed ASC in our experiments, where this treatment resulted in a significant increase in tumor progression compared to all other treatment groups evaluated.

## Data Availability

The data presented in the study are deposited in the NCBI GEO Datasets repository, accession number GSE245489.
