# Supplementary material for: Poly I:C-priming of adipose-derived mesenchymal stromal cells promotes a pro-tumorigenic phenotype in an immunocompetent mouse model of prostate cancer
Source: Front Cell Dev Biol. 2023 Nov 22;11:1145421. doi: 10.3389/fcell.2023.1145421 (PMC10703370; doi:10.3389/fcell.2023.1145421)
Supplement: Supplementary file 1 [file DataSheet1.zip › Supplementary Files/Supplementary File 1.pdf]

Sample QR code

Product Data Sheet

Product: 89-hASC

Donor ID: JW20150526

Product Description:

Organism: Human
Tissue:
Morphology:
Disease:

Cells/Vial: 1 million
Volume:
Appearance:
Sterility: Pass

Flow Cytometry (%) - P0			
CD29 PE	36.9	CD73 PE	20.83
CD105 PE	31.36	CD90 PE	38.8
CD45 PE	6.1	IgG PE	12.09
CD34 PE	10.62	IgG FITC	45.03
CD44 FITC	37.55		

Donor Demographics	
Gender	Male
Age	44
Race	Caucasian
BMI	30.04

Proliferation/CFU	
Proliferation doubling time (days)	2.1
CFU-F Percentage	N/A

Handling Procedure: *insert handling procedure*

Differentiation Images

Oil Red O-Control

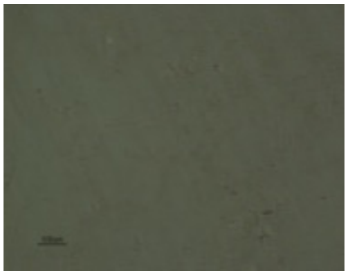

Oil Red O-Adipogenesis

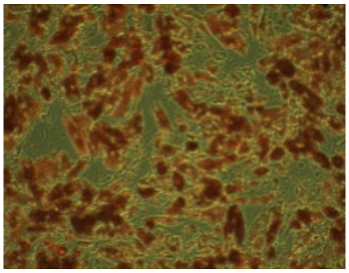

Alizarin Red-Control

Alizarin Red-Osteogenesis

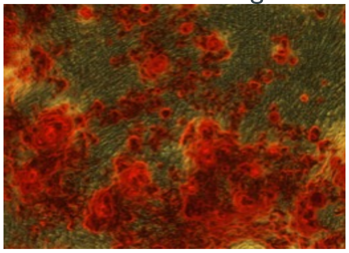

Obatala Sciences, Inc
2000 Lakeshore Dr., #4020
New Orleans, LA 70148

"Driving Diversity in Research.
Impacting the Future…"

Product Data Sheet

Storage Conditions

Intended Use

Certificate of Analysis
Here we would provide specific information related to each assay performed on the donor and the results

Growth Conditions
Temperature: 37oC
Atmosphere: 95% Air, 5% CO2

Seeding Density:

Recommended Media or ECM: We recommend using ObaGel® when culturing this model…(This will change depending on the cell type)

Material Citation
If use of this material results in a publication, please cite the material in the following manner…

References:

Additional Information

Warranty

Disclaimer

All Obatala Sciences Inc products and technology are GLP compliant and are to be used for research purposes only. Obacell™ products have been tested negative for HIV-1, HIV-2, Hepatitis B, and Hepatitis C.

Authorized Use of Obatala Sciences Inc Products

Products sold by Obatala Sciences Inc are to be handled by only personnel who have undergone proper laboratory compliance, including, but not limited to blood borne pathogen training. The training should be in strict accordance with all national, state, and local laws and regulations. By purchasing this product, you understand and accept these terms of sale.

Obatala Sciences, Inc
2000 Lakeshore Dr., #4020
New Orleans, LA 70148
